# CPAF: A Chlamydial Protease in Search of an Authentic Substrate

**DOI:** 10.1371/journal.ppat.1002842

**Published:** 2012-08-02

**Authors:** Allan L. Chen, Kirsten A. Johnson, Jennifer K. Lee, Christine Sütterlin, Ming Tan

**Affiliations:** 1 Department of Microbiology and Molecular Genetics, University of California at Irvine, Irvine, California, United States of America; 2 Department of Developmental and Cell Biology, University of California at Irvine, Irvine, California, United States of America; 3 Department of Medicine, University of California at Irvine, Irvine, California, United States of America; Yale University School of Medicine, United States of America

## Abstract

Bacteria in the genus *Chlamydia* are major human pathogens that cause an intracellular infection. A chlamydial protease, CPAF, has been proposed as an important virulence factor that cleaves or degrades at least 16 host proteins, thereby altering multiple cellular processes. We examined 11 published CPAF substrates and found that there was no detectable proteolysis when CPAF activity was inhibited during cell processing. We show that the reported proteolysis of these putative CPAF substrates was due to enzymatic activity in cell lysates rather than in intact cells. Nevertheless, *Chlamydia*-infected cells displayed *Chlamydia*-host interactions, such as Golgi reorganization, apoptosis resistance, and host cytoskeletal remodeling, that have been attributed to CPAF-dependent proteolysis of host proteins. Our findings suggest that other mechanisms may be responsible for these *Chlamydia*-host interactions, and raise concerns about all published CPAF substrates and the proposed roles of CPAF in chlamydial pathogenesis.

## Author Summary

*Chlamydia* are bacteria that invade eukaryotic host cells and live within a membrane-bound compartment called the chlamydial inclusion. Growth and survival of these important human and animal pathogens depends on extensive interactions with the host cell, which allow chlamydiae to acquire critical nutrients and to avoid host anti-microbial defenses. Chlamydiae are proposed to cause many of these host-pathogen interactions through the cleavage or degradation of host proteins by the chlamydial protease CPAF, which is secreted into the host cytoplasm. Here, we raise questions about the proposed roles of this virulence factor during infection, as well as its published substrates. We found that there was no detectable cleavage or degradation of 11 previously reported CPAF substrates in *Chlamydia*-infected cells and that CPAF-mediated proteolysis of these host proteins occurs during cell harvest and lysis. However, we still observed host-pathogen interactions previously attributed to CPAF proteolysis of these proteins, suggesting that *Chlamydia* is likely to cause these effects on the host cell through other mechanisms. Our findings call for a re-evaluation of all published CPAF substrates as well as the proposed roles of this protease in chlamydial pathogenesis.

## Introduction

*Chlamydia* are obligate intracellular bacteria that are responsible for more infections reported to the CDC than all other infectious agents combined [1]. *Chlamydia trachomatis* causes the most prevalent bacterial sexually transmitted disease in the United States [2] and the most common form of preventable blindness worldwide [3]. Another species, *Chlamydia pneumoniae*, is a causative agent of community-acquired pneumonia [4]. Despite this wide range of clinical manifestations, *Chlamydia* spp. display many similarities at the level of the intracellular infection. Chlamydiae replicate within a membrane-bound compartment called the chlamydial inclusion in which the bacterium converts between two specialized forms. During this developmental cycle, chlamydiae usurp or subvert a number of processes within the host cell to support the infection. For instance, *Chlamydia* alters the host secretory pathway to acquire lipids from post-Golgi vesicles to support growth of the inclusion and bacterial replication [5]–[7]. It also blocks host cell apoptosis, which could otherwise be used as a host defense mechanism against this intracellular pathogen that requires 2–3 days to complete its developmental cycle [8]–[10].

CPAF (*c*hlamydial *p*rotease or proteasome-like *a*ctivity *f*actor) has been proposed to be a major virulence factor in *Chlamydia*-infected cells [11]. This atypical serine protease [12] is conserved within the *Chlamydiales* [13], including the distantly related environmental chlamydiae, which include endosymbionts of amoeba [14]. During an infection, CPAF is secreted into the host cytoplasm where it has been reported to cleave or degrade specific host proteins [15]. A rapidly growing number of CPAF substrates has been reported [11], including at least 16 host proteins (Table 1).

**Table 1:** Summary of published CPAF substrates.

Reported Substrate	Reported Proteolysis	References	Proteolysis when CPAF is inhibited during cell processing (this study)
*Host Proteins*			
Golgin-84	Cleavage	[16]–[17]	No cleavage
Puma	Degradation	[18]–[19], [36], [48]	No degradation
Bim	Degradation	[18]–[19], [36], [48]	No degradation
Bik	Degradation	[19], [36]	No degradation
Keratin-8	Cleavage	[20], [30]	No cleavage
Keratin-18	Partial Cleavage	[20]	No cleavage
Vimentin	Partial Cleavage	[20]	No cleavage
p65/RelA	Cleavage	[22], [38]	No cleavage
Cyclin B1	Cleavage	[18], [31]	No cleavage
Nectin-1	Degradation	[23], [49]	No degradation
RFX5	Degradation	[15], [21]	No degradation
USF-1	Degradation	[15], [50]	Not tested
CD1d	Degradation	[51]	Not tested
PARP	Partial Cleavage	[18], [52]	Not tested
HMGB1	Cleavage	[52]	Not tested
HIF-1	Degradation	[53]	Not tested
*Chlamydial proteins*			
CPAF	Cleavage	[12], [32]	Cleavage
CT005	Degradation	[37]	Not tested
IncD (CT115)	Degradation	[37]	Not tested
IncE (CT116)	Cleavage	[37]	Not tested
IncC (CT233)	Degradation	[37]	Not tested
CT288	Degradation	[37]	Not tested
CT694	Degradation	[37]	Not tested
CT813	Cleavage	[37]	Not tested
TARP (CT456)	Degradation	[37]	Not tested

This proteolysis of host proteins by CPAF has been proposed to cause a number of effects on the infected host cell. For example, cleavage of the Golgi matrix protein golgin-84 has been reported to cause fragmentation and reorganization of the Golgi apparatus in *Chlamydia*-infected cells [16]–[17]. Similarly, degradation of pro-apoptotic BH3-only proteins, such as Puma, Bik, and Bim, has been proposed to mediate resistance to apoptosis [18]–[19]. Cleavage of several intermediate filaments has been linked to the dynamic remodeling of the host cytoskeleton around the growing chlamydial inclusion [20]. In addition, proteolysis of host transcription factors, such as the MHC transcription factor RFX5 [15], [21] and the p65/RelA subunit of NFκB [22], have been implicated as chlamydial strategies for evading the immune response of the host cell. In general, these associations have been inferred from the known functions of reported CPAF substrates, but the direct effects of these proteolytic events in *Chlamydia*-infected cells have not been examined [11].

The evidence for CPAF as a chlamydial protease that targets host proteins has been largely based on the observed cleavage or degradation of specific host proteins during an infection, as assayed by immunoblotting lysates from *Chlamydia*-infected cells [15]–[16], [18]–[20], [22]–[23]. In addition, many reports demonstrated that the timing of this proteolysis correlates with the expression of CPAF during the developmental cycle [24] and can be prevented *in vitro* [15], [19], [23] and *in vivo* [20], [23] by the CPAF inhibitor lactacystin [15]. Furthermore, the same cleavage or degradation patterns have been reproduced when recombinant CPAF was used *in vitro* [15], [19]–[20], [23] or overexpressed in uninfected cells [16], [18], [22].

In this study, we demonstrate that the reported cleavage or degradation of 11 published CPAF substrates was abrogated when the enzymatic activity of CPAF was inhibited during cell harvest and lysate preparation. However, we still observed host-pathogen interactions, such as Golgi reorganization and resistance to apoptosis, that are proposed to result from proteolysis of target proteins by CPAF. Our findings indicate that these host-*Chlamydia* interactions are likely to be mediated by mechanisms other than CPAF-dependent proteolysis of these host proteins. These results invite a reappraisal of previously identified CPAF substrates and re-interpretation of models involving the function of this chlamydial enzyme in the intracellular infection.

## Results

### Re-examination of Golgin-84 Cleavage during a Chlamydial Infection

**Figure 1:**
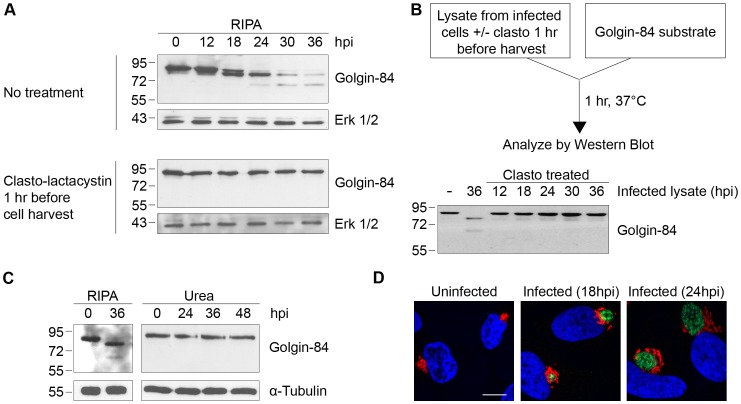
Golgin-84 cleavage does not occur in *Chlamydia*-infected cells when CPAF is inhibited during cell processing. (A) Uninfected (0 hpi) and infected cells at time points between 12 and 36 hpi were treated with methyl acetate as a solvent control (top panel) or 150 µM of the CPAF inhibitor *clasto*-lactacystin (bottom panel) for 1 hour prior to cell lysis in RIPA buffer. Total cell lysates were separated by SDS-PAGE and probed with antibodies against golgin-84 or Erk 1/2 (loading control). (B) Cell-free degradation assay testing for CPAF activity in lysates prepared from the *Chlamydia*-infected HeLa cells described in Figure 1A. Each infected cell lysate was incubated with a lysate of uninfected HeLa cells as the source of golgin-84 substrate and reactions were analyzed by immunoblotting with golgin-84 antibodies. (C) Lysates of uninfected (0 hpi) or infected cells from different times in the infection were prepared in RIPA buffer (left panel) or by direct lysis in 8M urea (right panel), separated by SDS-PAGE and analyzed with antibodies to golgin-84 or α-tubulin (loading control). (D) Confocal images of uninfected or *Chlamydia*-infected HeLa cells examined at 18 and 24 hpi. Cells were stained with antibodies to the Golgi marker α-mannosidase II (red), the chlamydial major outer membrane protein MOMP (green) and the DNA dye Hoechst 33342 (blue) to detect Golgi membranes, the chlamydial inclusion and DNA, respectively. Scale bar, 10 µm.

The Golgi protein golgin-84 is reported to be cleaved by CPAF in *Chlamydia*-infected cells [16], but we found that cleavage is dependent on the method of cell harvest and lysis (which we will hereafter collectively refer to as ‘cell processing’). To reproduce the published proteolysis, we harvested infected cells under standard lysis conditions in RIPA buffer [25] and analyzed the cell lysate by SDS-PAGE, followed by immunoblotting with anti-golgin-84 antibodies. Consistent with previous reports, there was progressive conversion of full-length golgin-84 into two cleavage products of ∼78 and ∼65 kDa beginning at 18 hours post infection (hpi) (Figure 1A, top panel), and most of the full-length protein in the extracts was cleaved by 36 hpi [16]–[17]. In striking contrast, there was no golgin-84 cleavage, even as late as 36 hpi, when we treated *Chlamydia*-infected cells for one hour prior to cell processing with 150 µM of *clasto*-lactacystin *β*-lactone, which is the active form of the CPAF inhibitor lactacystin [15], [26] (Figure 1A, compare top and bottom panels). This inhibition of golgin-84 proteolysis is unlikely to be due to the activity of *clasto*-lactacystin as a proteasome inhibitor [27] because we did not detect inhibition of proteasome function in our infected cells from the one hour treatment (data not shown). These results suggest that the cleavage of golgin-84 occurred during or after cell processing in standard lysis buffers and was prevented by inhibiting CPAF activity prior to these manipulations.

We next developed a cell-free assay to test for CPAF activity in lysates of *Chlamydia*-infected cells. When we incubated infected cell lysate with a lysate of uninfected HeLa cells as a source of golgin-84 substrate, there was almost complete cleavage of golgin-84 at 37°C (Figure 1B) and at 0°C (Figure S1A). These *in vitro* experiments demonstrate that CPAF remains active in lysates from *Chlamydia*-infected cells, even on ice, and thus could cleave a putative substrate during lysate preparation. In contrast, lysates of *Chlamydia*-infected cells pre-treated with *clasto*-lactacystin, for one hour before processing at times up to 36 hpi, did not cleave golgin-84 in this *in vitro* assay (Figure 1B). These results show that CPAF activity during lysate preparation can be abolished by treating the infected cells with *clasto*-lactacystin for one hour prior to cell processing.

To determine whether *clasto*-lactacystin was preventing golgin-84 cleavage during the one hour treatment before cell lysis or during cell processing itself, we used an alternative approach to inhibit CPAF activity during cell processing. In these experiments, we lysed cells in urea as a denaturing agent to block enzymatic activity in our lysates [28]. When we lysed *Chlamydia*-infected cells by adding 8M urea directly to the monolayer, no golgin-84 cleavage was observed even as late as 48 hpi (Figure 1C). These lysates did not contain detectable CPAF activity as verified with the cell-free degradation assay (data not shown). This lack of golgin-84 cleavage was observed for *Chlamydia* infection of HeLa cells and two other human cell lines when lysed in urea (Figure S1B). Taken together, these results lead us to conclude that the reported CPAF-dependent cleavage of golgin-84 is unlikely to occur in intact cells. Our results are consistent with an explanation that proteolysis occurred during cell processing and is due to CPAF activity in the lysates of *Chlamydia*-infected cells.

We examined Golgi organization in cells on parallel coverslips because cleavage of golgin-84 has been proposed to induce Golgi fragmentation in *Chlamydia*-infected cells [17]. In uninfected control cells, Golgi membranes were arranged as an interconnected Golgi ribbon in the pericentriolar region, as detected by immunofluorescence with antibodies to the Golgi marker mannosidase II [29]. In contrast, Golgi membranes of infected cells were reorganized around the growing chlamydial inclusion by 18 and 24 hpi (Figure 1D), which is consistent with previous reports [17]. Thus, Golgi reorganization in our *Chlamydia*-infected cells occurs in the absence of detectable golgin-84 cleavage and is unlikely to be caused by CPAF-dependent proteolysis of this structural Golgi protein.

### Degradation of Pro-apoptotic BH3-only Proteins Is Also Dependent on Cell Processing

**Figure 2:**
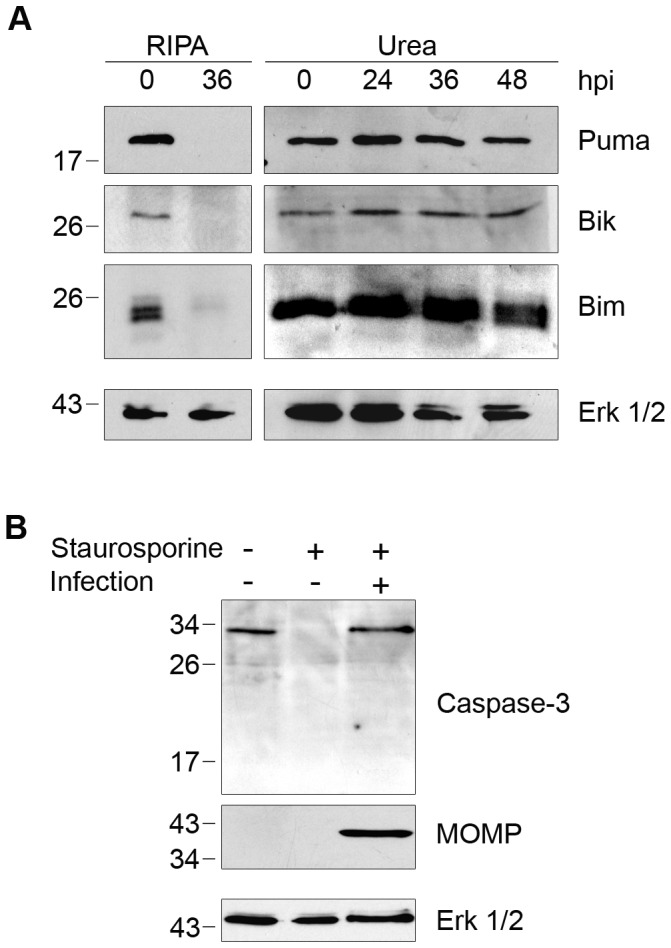
Degradation of BH3-only proteins in *Chlamydia*-infected cells is prevented by inhibiting CPAF during cell processing. (A) Lysates of uninfected (0 hpi) or infected HeLa cells were prepared in RIPA buffer (left panel) or by direct lysis in 8M urea (right panel) at the indicated times, separated by SDS-PAGE and analyzed by immunoblotting with antibodies to the proapoptotic BH3-only proteins Puma, Bik, or Bim. Equal loading was monitored for each blot with antibodies to Erk 1/2, but only the loading control for the Puma blot is shown as an example. (B) Uninfected or *Chlamydia*-infected HeLa cells were treated with 1 µM staurosporine to induce apoptosis, which was monitored by the loss of full-length caspase-3. Immunoblots of the lysates were probed with antibodies to caspase-3, MOMP (marker of *Chlamydia* infection), or Erk 1/2 (loading control).

Our findings with golgin-84 motivated us to investigate other host proteins reported to be CPAF substrates based on immunoblots of lysates prepared with standard buffers. The resistance of *Chlamydia-*infected cells to apoptosis [8]–[10] has been proposed to be mediated by CPAF-dependent degradation of BH3-only proteins, including Puma, Bik, and Bim [18]–[19]. We again replicated the complete degradation of each of these proteins by lysing *Chlamydia*-infected cells in RIPA buffer at 36 hpi (Figure 2A). However, when infected cells were lysed in 8M urea, Puma, Bik, and Bim were unaltered even at 48 hpi (Figure 2A). Thus, as with cleavage of golgin-84, inhibition of CPAF activity during cell processing abolished the published degradation of these pro-apoptotic factors.

Although Puma, Bik, and Bim were not degraded in *Chlamydia*-infected cells, these cells were resistant to staurosporine-induced programmed cell death, as previously reported [8]. Staurosporine treatment for 3 hours caused the complete loss of full-length caspase-3 in uninfected cells, but there was no decrease in full-length caspase-3 levels in our *C. trachomatis*-infected cells (Figure 2B). Based on these results, it is doubtful that the anti-apoptotic effects of a chlamydial infection on the host cell can be attributed to CPAF-dependent degradation of the BH3-only proteins Puma, Bik, and Bim.

### Proteolysis of Seven Additional Host Proteins by CPAF Is Dependent on Cell Processing

**Figure 3:**
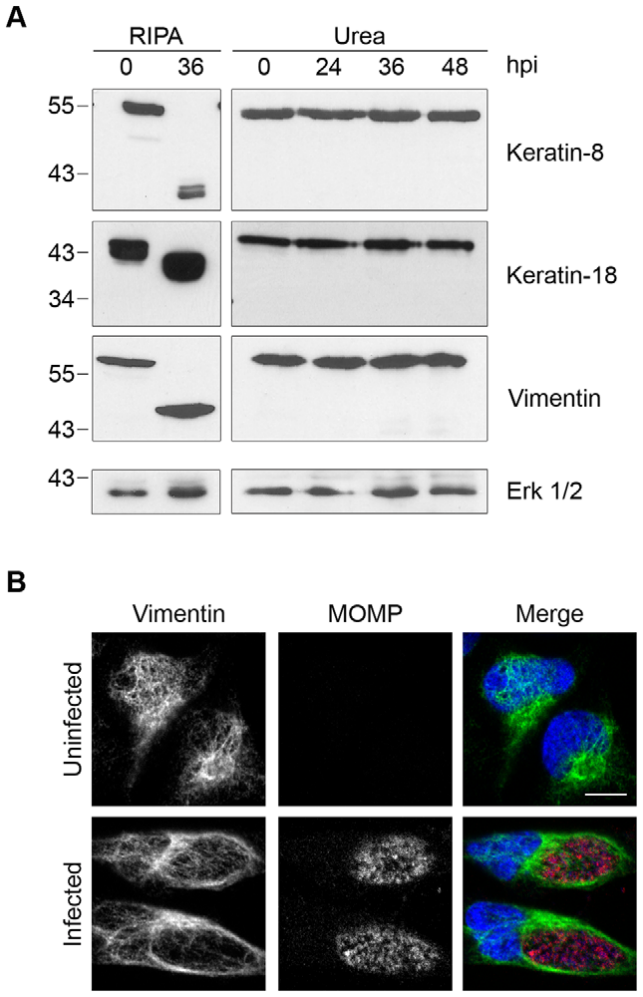
Cleavage of intermediate filaments in *Chlamydia*-infected cells is also dependent on cell processing. (A) Lysates of uninfected (0 hpi) or infected HeLa cells were prepared in RIPA buffer (left panel) or by direct lysis in 8M urea (right panel) at the indicated times, separated by SDS-PAGE and analyzed by immunoblotting with antibodies to keratin-8, keratin-18, or vimentin. Equal loading for each blot was monitored by blotting for Erk 1/2 (loading control), but only the loading control for keratin-8 is shown. (B) Uninfected and infected HeLa cells at 30 hpi were fixed and stained with antibodies to vimentin (green), the chlamydial major outer membrane protein MOMP (red) and the DNA dye Hoechst 33342 (blue). Representative confocal images are shown. Scale bar, 10 µm.

We next examined the proteolytic effects of CPAF on the three intermediate filaments keratin-8, keratin-18, and vimentin, whose cleavage has been implicated in the growth of the chlamydial inclusion [20], [30]. When we lysed *Chlamydia*-infected cells at 36 hpi in RIPA buffer (Figure 3A), we observed the conversion of each full-length protein into smaller fragments, as previously reported [20], [30]. In contrast, when we lysed cells directly in 8M urea, we did not detect any proteolytic effects on these proteins over the time course of an infection up to 48 hpi (Figure 3A), indicating that CPAF was unlikely to cleave these proteins in intact cells. CPAF-dependent cleavage of vimentin has been proposed to be important for host cytoskeletal rearrangement into a supportive cage surrounding the chlamydial inclusion [20]. However, we still observed a cage-like vimentin structure around the inclusion in our *Chlamydia*-infected cells (Figure 3B), which suggests that this reorganization of the host cytoskeleton during an infection does not require vimentin cleavage.

**Figure 4:**
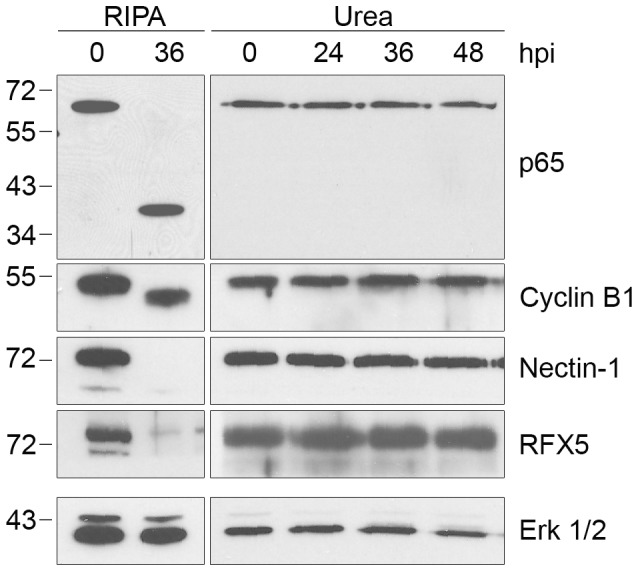
Proteolysis of four additional CPAF substrates is dependent on cell processing. Lysates of uninfected (0 hpi) or infected HeLa cells were prepared in RIPA buffer (left panel) or by direct lysis in 8M urea (right panel) at the indicated times, separated by SDS-PAGE and probed with antibodies to the p65/RelA subunit of NFκB, cyclin B1, nectin-1, or RFX5 as indicated. Equal loading for each blot was monitored by blotting for Erk 1/2, but only the loading control for nectin-1 is shown.

We also examined proteolytic effects of CPAF on four additional host proteins that have been reported to be CPAF substrates. These include the NFκB transcription factor subunit p65/RelA [22], the MHC transcription factor RFX5 [15], [21], the adherens junction protein nectin-1 [23], and the cell cycle protein cyclin B1 [18], [31]. As with the other substrates analyzed, the apparent proteolysis of these four proteins was only observed when *Chlamydia*-infected cells were lysed in RIPA buffer and not with direct lysis in urea (Figure 4). Taken together, these studies demonstrate that the previously reported proteolysis of 11 published CPAF substrates can be prevented by inhibiting CPAF activity during the processing of *Chlamydia*-infected cells (Table 1).

### Evidence That There Is CPAF Activity in *Chlamydia*-Infected Cells

**Figure 5:**
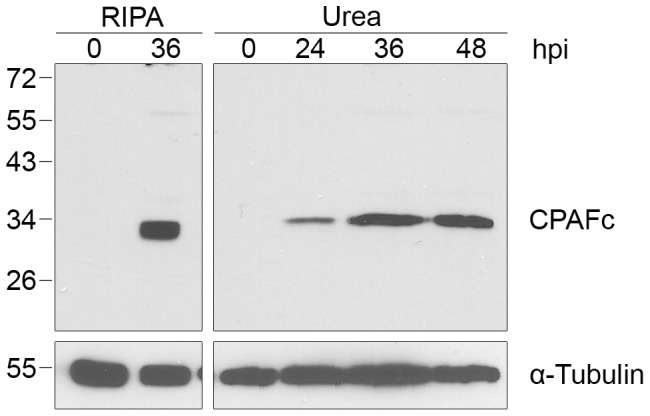
CPAF is autocatalytically cleaved into its active form in *Chlamydia-*infected cells. Lysates of uninfected (0 hpi) or infected HeLa cells were prepared in RIPA buffer (left panel) or by direct lysis in 8M urea (right panel) at the indicated times, separated by SDS-PAGE and probed with antibodies to the C-terminal fragment of CPAF (CPAFc) and α-tubulin (loading control).

Since we did not detect CPAF activity towards reported host substrates when we lysed cells in 8M urea, we examined if CPAF itself was cleaved into its enzymatically-active form in our *Chlamydia*-infected cells [12], [32]. CPAF is synthesized as a zymogen of 70 kDa that is converted in *trans* into active N- and C-terminal fragments via an autocatalytic cleavage reaction that requires CPAF proteolytic activity [12], [32]–[35]. Using an antibody that recognizes the C-terminal fragment of CPAF, CPAFc [35], we detected only this cleaved form but not the full-length zymogen in *Chlamydia*-infected cell lysates prepared with either RIPA buffer or 8M urea (Figure 5). This C-terminal fragment of CPAF accumulated over the course of infection from 24 to 48 hpi (Figure 5). By verifying CPAF as its own substrate, we provide evidence of CPAF catalytic activity in *Chlamydia*-infected cells even under our most stringent cell processing conditions.

## Discussion

We have re-examined the reported proteolysis of specific host proteins by the enzyme CPAF in *Chlamydia*-infected cells. Specifically, we show that cleavage or degradation of 11 independent host proteins was abrogated by treating cells with the CPAF inhibitor *clasto*-lactacystin for one hour prior to cell processing or by lysing cells from a monolayer directly in the denaturing agent urea. A major strength of our study is that we can reproduce the reported proteolytic effects of CPAF under standard lysis conditions and then prevent these effects solely by altering the conditions used to process the cells for protein analysis. In addition, there was no detectable CPAF-dependent proteolysis up to 48 hpi, which is many hours after the substrates are reported to be cleaved or degraded [16]–[18], [20], [30], [36]. These surprising results raise questions about the identity of authentic CPAF substrates and the timing and significance of their proteolysis during a *Chlamydia* infection.

Several lines of evidence have been used to identify putative CPAF substrates. The cleavage or degradation of specific host proteins has been detected by immunoblot analyses of lysates from *Chlamydia*-infected cells prepared by standard lysis procedures [15]–[16], [18]–[20], [22]–[23]. These proteolysis patterns have been reproduced *in vitro* by recombinant CPAF [15], [19]–[20], [23] and *in vivo* by overexpression of CPAF in uninfected cells [16], [18], [22], suggesting that CPAF may be the cognate protease. The involvement of this chlamydial protease in these cleavage reactions has been further supported by data showing that proteolysis of specific substrates can be prevented *in vitro* by a CPAF inhibitor, such as lactacystin [15], [19], [23]. However, only a few studies have provided evidence of proteolysis in intact infected cells with techniques such as immunofluorescence [23].

How can we reconcile our results with the large body of published data on CPAF and its substrates? Our studies demonstrate that there is CPAF activity in lysates of *Chlamydia*-infected cells prepared under standard conditions (i.e. lysis of cells in RIPA buffer in the presence of a standard protease inhibitor cocktail on ice). CPAF may remain active in the lysate because it is an atypical serine protease that is not inhibited by a range of protease inhibitors [21], [37]. This enzymatic activity can then cleave or degrade susceptible host proteins in the lysate, even when left on ice, because CPAF is active at 0°C (Figure S1A). When we prevented CPAF activity in lysates obtained from monolayers of *Chlamydia*-infected cells, we no longer observed proteolysis of these 11 host proteins as late as 48 hpi. These findings lead us to conclude that the published cleavage and degradation of these host proteins in *Chlamydia*-infected cells are unlikely to have occurred in intact cells but instead are due to *in vitro* proteolysis by CPAF during cell processing. Our results do not rule out the possibility that there could be a small amount of cleavage or degradation of one or more of these purported substrates below the detection limit of immunoblots. In addition, the specificity with which these proteins are cleaved or degraded by CPAF *in vitro* suggests that they have the potential to be *in vivo* CPAF substrates under conditions in a *Chlamydia*-infected cell that have not yet been elucidated.

These findings emphasize the importance of monitoring CPAF activity during cell processing in order to distinguish between proteolytic activity *in vitro* and *in vivo.* Since we did not detect proteolytic effects for any of the 11 published host substrates in this study, the proteolysis of other reported CPAF substrates should be re-examined to verify that they are altered *in vivo*. However, there is evidence of CPAF enzymatic activity within a *Chlamydia*-infected cell because CPAF itself was cleaved even though we inhibited CPAF activity during cell harvest and lysis (Figure 5). This cleavage of CPAF is consistent with its autocatalytic processing [34].

The multiple proposed roles of CPAF in mediating host-pathogen interactions during a chlamydial infection merit reappraisal. Our data demonstrate that *Chlamydia*-induced Golgi reorganization occurs in the absence of detectable golgin-84 cleavage. Also, resistance of *Chlamydia*-infected cells to apoptosis is unlikely to be caused by the degradation of the pro-apoptotic BH3-only proteins Puma, Bik, and Bim. Similarly, the cage-like vimentin structure surrounding the chlamydial inclusion was present even though vimentin cleavage was not evident. It has been reported that NFκB activation is blocked in *Chlamydia*-infected cells by cleavage of p65/RelA by either CPAF [22], [38] or another chlamydial protease CT441/Tsp [22], [38]. Our results indicate that this controversy may be moot because we did not detect any appreciable cleavage of p65/RelA. These proposed effects of CPAF have been largely based on the proteolysis of specific host proteins and inferred from the known functions of these proteins in an uninfected cell [18]–[20]. However, there has been little direct evidence showing that CPAF-mediated proteolysis is necessary for the specific host-pathogen interactions during a chlamydial infection. In the absence of detectable CPAF-dependent proteolysis of golgin-84, the three BH3-only proteins, vimentin, and p65/RelA, these host-*Chlamydia* interactions are probably mediated by alternative mechanisms. For example, it has been proposed that resistance of *Chlamydia*-infected cells to apoptosis is due to upregulation of the anti-apoptotic factors cIAP-2 and Mcl-1 and not the degradation of BH3-only proteins [39]–[40].

Despite these questions about CPAF, this conserved chlamydial protease is likely to play an important role during the intracellular chlamydial infection. We have shown that CPAF is still cleaved into its active form (Figure 5), suggesting that it can potentially cleave or degrade host targets in an infected cell. Moreover, treatment of infected cells with CPAF inhibitors can prevent normal chlamydial growth and development [16], [37]. However, in light of the current findings, the exact role and timing of CPAF activity in a *Chlamydia*-infected cell remains to be determined.

Our studies on CPAF have general implications for the analysis of modification enzymes such as kinases or proteases that may retain activity under standard cell processing conditions. This concern is most obvious for proteases that are resistant to standard protease inhibitor cocktails. For example, hundreds of caspase substrates have been identified, but many are considered “bystander” substrates that are not biologically relevant even though they contain specific cleavage sites for these proteases [41]. Our findings underscore the importance of inhibiting the relevant enzymatic activity during cell processing and verifying that this activity is actually blocked.

This study highlights the need to re-evaluate the *Chlamydia* literature on CPAF. General concerns have been raised about continued CPAF activity during cell lysis [11], [31], but the present study shows that at least 11 of 16 previously identified host CPAF substrates are not observed to be altered during the normal course of infection. Biochemical reports about the activation of CPAF [12], [32]–[35], and studies characterizing antibody responses to CPAF [42]–[45], are likely to be unaffected by our findings. However, the presence of CPAF activity under standard lysis conditions calls for a reassessment of prior studies on CPAF substrates and the role of CPAF in *Chlamydia*-infected cells.

## Materials and Methods

### Antibodies

The following antibodies were used in this study: rabbit anti-Bim, rabbit anti-Puma, mouse anti-keratin-8, mouse anti-keratin-18, mouse anti-α-tubulin, and mouse anti-vimentin (all from Sigma-Aldrich); rabbit anti-cyclin B1, mouse anti-nectin-1, mouse anti-p65/RelA and bovine anti-sheep-HRP (all from Santa Cruz Biotechnology); rabbit anti-Bik, rabbit anti-vimentin, and mouse anti-Erk 1/2 (all from Cell Signaling Technology); goat anti-mouse-HRP and goat anti-rabbit-HRP (both from Jackson ImmunoResearch Laboratories); mouse anti-caspase-3 (BD Biosciences); rabbit anti-RFX5 (Rockland Immunochemicals); Alexa Fluor 488 or Alexa Fluor 594-tagged goat anti-mouse or goat anti-rabbit antibodies (Molecular Probes/Invitrogen); mouse anti-CPAFc (generous gift from Dr. Guangming Zhong, University of Texas Health Science Center at San Antonio); sheep anti-golgin-84 (kindly provided by Dr. Martin Lowe, University of Manchester); rabbit anti-α-mannosidase II (kindly provided by Dr. Kelley Moremen, University of Georgia); mouse anti-MOMP (VD4 epitope of the major outer membrane protein from *C. trachomatis* serovar E [BOUR]) (kind gift from Dr. Ellena Peterson, UC Irvine).

### Cell Culture

HeLa cells (ATCC) were grown in 6-well dishes in Advanced DMEM (4.5 g glucose/L) (Invitrogen) supplemented with 2% fetal bovine serum (FBS) (Hyclone/Thermo Fisher) and 2 mM GlutaMAX-I (Invitrogen). HEK 293T cells and retinal pigment epithelial (hTERT RPE-1) cells (both from ATCC) were cultured in DMEM (4.5 g glucose/L) (Invitrogen) supplemented with 10% fetal bovine serum. All cell lines were grown in 5% CO_2_ at 37°C and screened for *Mycoplasma* contamination by PCR [46].

### *Chlamydia* Infections

Cell monolayers were infected with *C. trachomatis* serovar L2 (L2/434/Bu), LGV biovar, at a multiplicity of infection (MOI) of 3 in sucrose-phosphate-glutamic acid (SPG). In parallel, uninfected control experiments were performed as mock infections in SPG alone. Infections were carried out by centrifugation at 700× *g* in a Sorvall Legend Mach 1.6R centrifuge for 1 hr at room temperature. After centrifugation, the inoculum was replaced by fresh cell culture medium and monolayers were incubated at 37°C and 5% CO_2_. Chlamydial EBs (elementary bodies) were verified to be free of *Mycoplasma* contamination by PCR [46].

### Cell Processing and Immunoblotting

Lysis in RIPA buffer: Cells were harvested by trypsinization for 3–5 min at 37°C and the trypsinized cells were transferred to a 15 mL conical tube on ice. The dish was washed twice with 1×PBS and the washes were added to the 15 mL conical tube to collect any remaining cells. The cells were pelleted by centrifugation at 1500 rpm for 3 min at 4°C and lysed on ice for 10 min in RIPA buffer (50 mM Tris [pH 7.5], 150 mM NaCl, 0.1% SDS, 0.5% sodium deoxycholate, 1% NP-40) supplemented with protease inhibitors (2 mM pepstatin, 150 mM aprotinin [both from MP Biochemicals], 1 mM leupeptin [Calbiochem], 1 mM PMSF [Acros]). The cells were resuspended by pipetting up and down in approximately 1 mL of ice-cold lysis buffer per 5×10^6^ cells. Lysates were cleared by centrifugation at 13,000× *g* for 10 min at 4°C and protein concentrations were determined by Bradford assay (BioRad).

Lysis in urea: A solution of 8M urea was supplemented with 325 U/mL of Benzonase Nuclease (Sigma-Aldrich) and added directly to cell monolayers at a volume of 1 mL per 6-well dish for 10 min on ice. Lysates were then pooled and protein concentrations were determined by the DC protein assay (BioRad).

Cell lysates were diluted into Laemmli sample buffer (50 mM Tris-HCl [pH 6.8], 10% glycerol, 2% SDS, 1% 2-mercaptoethanol, 0.1% bromophenol blue). Samples containing equal amounts of protein were loaded and resolved by SDS-PAGE. Proteins were transferred onto nitrocellulose membranes and subjected to immunoblot analysis (Table S1) with enhanced chemiluminescence (90 mM p-Courmaric acid, 250 mM 3-Aminophthalhydrazide, 100 mM Tris-HCl [pH 8.5]).

*Clasto*-lactacystin treatment: 150 µM of *clasto*-lactacystin *β*-lactone (Cayman Chemical), dissolved in methyl acetate, was added to the cell culture medium for 1 hr prior to cell processing. For example, samples of *Chlamydia*-infected cells at 36 hpi were treated with *clasto*-lactacystin at 35 hpi for 1 hr and then processed. In parallel control experiments, methyl acetate as the solvent was added to the culture medium.

### Immunofluorescence

Cells grown on glass coverslips were fixed in 4% formaldehyde for 10 min at room temperature and blocked in 5% blocking buffer (0.1% Triton X-100, 5% FBS in PBS) or TBS-BSA (0.1% Tween-20, 5% BSA in TBS) for 1 hr. Cells were incubated with primary antibodies for 1 hr at room temperature or overnight at 4°C followed by Alexa-fluorochrome-conjugated secondary antibodies for 30 min at room temperature. Host and chlamydial DNA were stained with Hoechst 33342 (Molecular Probes/Invitrogen). Coverslips were mounted onto glass slides with gelvatol [47] and imaged by confocal microscopy on a Nikon Eclipse Ti-U inverted microscope fitted with a Nikon D-Eclipse confocal laser assembly and a D-Eclipse C1 controller (Nikon). Images were acquired using the Nikon EZ-C1 program and analyzed using Nikon NIS Elements and Adobe Photoshop.

### Cell-free Degradation Assays

*Chlamydia*-infected HeLa cells at various times in the infection were either left untreated, mock-treated with methyl acetate, or treated for 1 hr with 150 µM of *clasto*-lactacystin, and then lysed in RIPA buffer as described above. 3.5 µg of *Chlamydia*-infected HeLa cell lysate, as the source of CPAF, was incubated with 27 µg of uninfected HeLa cell lysate, as the source of host protein substrates, at 37°C for 1 hr in CPAF reaction buffer (25 mM Tris [pH 8.0], 150 mM NaCl, 3 mM DTT). For reactions performed at 0°C, 54 µg of *Chlamydia*-infected HeLa cell lysate from 36 hpi was incubated with 54 µg of uninfected HeLa cell lysate on ice for 10 or 30 min. Reactions were terminated by adding Laemmli sample buffer and boiling for 5 min.

### Apoptosis Induction Assay

Uninfected or *Chlamydia*-infected HeLa cells (MOI of 3, at 24 hpi) were incubated with 1 µM staurosporine in tissue culture medium for 3 hrs. Lysates of the cell monolayers were prepared by direct lysis in 8M urea as previously described, separated by SDS-PAGE and analyzed by immunoblotting with antibodies to caspase-3.

## Supporting Information

Figure S1 **Controls for golgin-84 cleavage experiments in** **Figure 1** **.** (A) CPAF is active at 0°C. Cell-free degradation assay in which *Chlamydia*-infected cell lysate as a source of CPAF was incubated on ice with uninfected cell lysate, as the source of host protein substrates. Reactions were incubated at 0°C for the times indicated and analyzed by immunoblotting with antibodies to golgin-84. (B) Golgin-84 is not cleaved in other *Chlamydia*-infected cell lines. Lysates of uninfected (0 hpi) or infected HEK 293T (labeled as 293T) and hTERT RPE-1 (labeled as RPE1) cells were prepared in RIPA buffer (left panel) or by direct lysis in 8M urea (right panel) at the indicated times, separated by SDS-PAGE and probed with antibodies to golgin-84 or α-tubulin (loading control).
(TIF)

Table S1 **Antibody information.**
(DOC)

## References

[1] CDC (2011). Summary of notifiable diseases: United States, 2009.. MMWR Morb Mortal Wkly Rep.

[2] Schachter J, Stephens RS (1999). Infection and disease epidemiology.. *Chlamydia*: Intracellular Biology, Pathogenesis, and Immunity.

[3] Burton MJ, Mabey DC (2009). The global burden of trachoma: a review.. PLoS Negl Trop Dis.

[4] Blasi F, Tarsia P, Aliberti S (2009). *Chlamydophila pneumoniae*.. Clin Microbiol Infect.

[5] Carabeo RA, Mead DJ, Hackstadt T (2003). Golgi-dependent transport of cholesterol to the *Chlamydia trachomatis* inclusion.. Proc Natl Acad Sci U S A.

[6] Hackstadt T, Rockey D, Heinzen R, Scidmore M (1996). *Chlamydia trachomatis* interrupts an exocytic pathway to acquire endogenously synthesized sphingomyelin in transit from the Golgi apparatus to the plasma membrane.. EMBO J.

[7] Hackstadt T, Scidmore MA, Rockey DD (1995). Lipid metabolism in *Chlamydia trachomatis*-infected cells: directed trafficking of Golgi-derived sphingolipids to the chlamydial inclusion.. Proc Natl Acad Sci U S A.

[8] Fan T, Lu H, Hu H, Shi L, McClarty GA (1998). Inhibition of apoptosis in *Chlamydia*-infected cells: blockade of mitochondrial cytochrome c release and caspase activation.. J Exp Med.

[9] Fischer SF, Schwarz C, Vier J, Hacker G (2001). Characterization of antiapoptotic activities of *Chlamydia pneumoniae* in human cells.. Infect Immun.

[10] Rajalingam K, Al-Younes H, Muller A, Meyer TF, Szczepek AJ (2001). Epithelial cells infected with Chlamydophila pneumoniae (Chlamydia pneumoniae) are resistant to apoptosis.. Infect Immun.

[11] Zhong G (2009). Killing me softly: chlamydial use of proteolysis for evading host defenses.. Trends Microbiol.

[12] Huang Z, Feng Y, Chen D, Wu X, Huang S (2008). Structural basis for activation and inhibition of the secreted *Chlamydia* protease CPAF.. Cell Host Microbe.

[13] Dong F, Zhong Y, Arulanandam B, Zhong G (2005b). Production of a proteolytically active protein, chlamydial protease/proteasome-like activity factor, by five different *Chlamydia* species.. Infect Immun.

[14] Horn M, Collingro A, Schmitz-Esser S, Beier CL, Purkhold U (2004). Illuminating the evolutionary history of chlamydiae.. Science.

[15] Zhong G, Fan P, Ji H, Dong F, Huang Y (2001). Identification of a chlamydial protease-like activity factor responsible for the degradation of host transcription factors.. J Exp Med.

[16] Christian JG, Heymann J, Paschen SA, Vier J, Schauenburg L (2011). Targeting of a chlamydial protease impedes intracellular bacterial growth.. PLoS Pathog.

[17] Heuer D, Rejman Lipinski A, Machuy N, Karlas A, Wehrens A (2009). *Chlamydia* causes fragmentation of the Golgi compartment to ensure reproduction.. Nature.

[18] Paschen SA, Christian JG, Vier J, Schmidt F, Walch A (2008). Cytopathicity of *Chlamydia* is largely reproduced by expression of a single chlamydial protease.. J Cell Biol.

[19] Pirbhai M, Dong F, Zhong Y, Pan KZ, Zhong G (2006). The secreted protease factor CPAF is responsible for degrading pro-apoptotic BH3-only proteins in *Chlamydia trachomatis*-infected cells.. J Biol Chem.

[20] Kumar Y, Valdivia RH (2008). Actin and intermediate filaments stabilize the *Chlamydia trachomatis* vacuole by forming dynamic structural scaffolds.. Cell Host Microbe.

[21] Zhong G, Liu L, Fan T, Fan P, Ji H (2000). Degradation of transcription factor RFX5 during the inhibition of both constitutive and interferon gamma-inducible major histocompatibility complex class I expression in *Chlamydia*-infected cells.. J Exp Med.

[22] Christian J, Vier J, Paschen SA, Hacker G (2010). Cleavage of the NF-kappaB family protein p65/RelA by the chlamydial protease-like activity factor (CPAF) impairs proinflammatory signaling in cells infected with Chlamydiae.. J Biol Chem.

[23] Sun J, Schoborg RV (2009). The host adherens junction molecule nectin-1 is degraded by chlamydial protease-like activity factor (CPAF) in *Chlamydia trachomatis*-infected genital epithelial cells.. Microbes Infect.

[24] Belland RJ, Zhong G, Crane DD, Hogan D, Sturdevant D (2003). Genomic transcriptional profiling of the developmental cycle of *Chlamydia trachomatis*.. Proc Natl Acad Sci U S A.

[25] Holden P, Horton WA (2009). Crude subcellular fractionation of cultured mammalian cell lines.. BMC Res Notes.

[26] Dick LR, Cruikshank AA, Destree AT, Grenier L, McCormack TA (1997). Mechanistic studies on the inactivation of the proteasome by lactacystin in cultured cells.. J Biol Chem.

[27] Fenteany G, Standaert RF, Lane WS, Choi S, Corey EJ (1995). Inhibition of proteasome activities and subunit-specific amino-terminal threonine modification by lactacystin.. Science.

[28] Rajagopalan KV, Fridovich I, Handler P (1961). Competitive inhibition of enzyme activity by urea.. J Biol Chem.

[29] Takizawa PA, Yucel JK, Veit B, Faulkner DJ, Deerinck T (1993). Complete vesiculation of Golgi membranes and inhibition of protein transport by a novel sea sponge metabolite, ilimaquinone.. Cell.

[30] Dong F, Su H, Huang Y, Zhong Y, Zhong G (2004c). Cleavage of host keratin 8 by a *Chlamydia*-secreted protease.. Infect Immun.

[31] Balsara ZR, Misaghi S, Lafave JN, Starnbach MN (2006). *Chlamydia trachomatis* infection induces cleavage of the mitotic cyclin B1.. Infect Immun.

[32] Dong F, Pirbhai M, Zhong Y, Zhong G (2004a). Cleavage-dependent activation of a *Chlamydia*-secreted protease.. Mol Microbiol.

[33] Chen D, Chai J, Hart PJ, Zhong G (2009). Identifying catalytic residues in CPAF, a *Chlamydia*-secreted protease.. Arch Biochem Biophys.

[34] Chen D, Lei L, Flores R, Huang Z, Wu Z (2010). Autoprocessing and self-activation of the secreted protease CPAF in *Chlamydia*-infected cells.. Microb Pathog.

[35] Dong F, Sharma J, Xiao Y, Zhong Y, Zhong G (2004b). Intramolecular dimerization is required for the *Chlamydia*-secreted protease CPAF to degrade host transcriptional factors.. Infect Immun.

[36] Dong F, Pirbhai M, Xiao Y, Zhong Y, Wu Y (2005a). Degradation of the proapoptotic proteins Bik, Puma, and Bim with Bcl-2 domain 3 homology in *Chlamydia trachomatis*-infected cells.. Infect Immun.

[37] Jorgensen I, Bednar MM, Amin V, Davis BK, Ting JP (2011). The *Chlamydia* protease CPAF regulates host and bacterial proteins to maintain pathogen vacuole integrity and promote virulence.. Cell Host Microbe.

[38] Lad SP, Li J, da Silva Correia J, Pan Q, Gadwal S (2007). Cleavage of p65/RelA of the NF-kappaB pathway by *Chlamydia*.. Proc Natl Acad Sci U S A.

[39] Rajalingam K, Sharma M, Lohmann C, Oswald M, Thieck O (2008). Mcl-1 is a key regulator of apoptosis resistance in *Chlamydia trachomatis*-infected cells.. PLoS One.

[40] Rajalingam K, Sharma M, Paland N, Hurwitz R, Thieck O (2006). IAP-IAP complexes required for apoptosis resistance of *C. trachomatis*-infected cells.. PLoS Pathog.

[41] Fischer U, Janicke RU, Schulze-Osthoff K (2003). Many cuts to ruin: a comprehensive update of caspase substrates.. Cell Death Differ.

[42] Cong Y, Jupelli M, Guentzel MN, Zhong G, Murthy AK (2007). Intranasal immunization with chlamydial protease-like activity factor and CpG deoxynucleotides enhances protective immunity against genital *Chlamydia muridarum* infection.. Vaccine.

[43] Murthy AK, Cong Y, Murphey C, Guentzel MN, Forsthuber TG (2006). Chlamydial protease-like activity factor induces protective immunity against genital chlamydial infection in transgenic mice that express the human HLA-DR4 allele.. Infect Immun.

[44] Sharma J, Bosnic AM, Piper JM, Zhong G (2004). Human antibody responses to a *Chlamydia*-secreted protease factor.. Infect Immun.

[45] Skwor T, Kandel RP, Basravi S, Khan A, Sharma B (2010). Characterization of humoral immune responses to chlamydial HSP60, CPAF, and CT795 in inflammatory and severe trachoma.. Invest Ophthalmol Vis Sci.

[46] Ossewaarde J, de Vries A, Bestebroer T, Angulo A (1996). Application of a *Mycoplasma* group-specific PCR for monitoring decontamination of *Mycoplasma*-infected *Chlamydia* sp. strains.. Appl Environ Microbiol.

[47] Harlow E, Lane D (1999). Using antibodies: a laboratory manual.

[48] Fischer SF, Vier J, Kirschnek S, Klos A, Hess S (2004). Chlamydia inhibit host cell apoptosis by degradation of proapoptotic BH3-only proteins.. J Exp Med.

[49] Sun J, Kintner J, Schoborg RV (2008). The host adherens junction molecule nectin-1 is downregulated in Chlamydia trachomatis-infected genital epithelial cells.. Microbiology.

[50] Zhong G, Fan T, Liu L (1999). Chlamydia inhibits interferon gamma-inducible major histocompatibility complex class II expression by degradation of upstream stimulatory factor 1.. J Exp Med.

[51] Kawana K, Quayle AJ, Ficarra M, Ibana JA, Shen L (2007). CD1d degradation in Chlamydia trachomatis-infected epithelial cells is the result of both cellular and chlamydial proteasomal activity.. J Biol Chem.

[52] Yu H, Schwarzer K, Forster M, Kniemeyer O, Forsbach-Birk V (2010). Role of high-mobility group box 1 protein and poly(ADP-ribose) polymerase 1 degradation in Chlamydia trachomatis-induced cytopathicity.. Infect Immun.

[53] Rupp J, Gieffers J, Klinger M, van Zandbergen G, Wrase R (2007). Chlamydia pneumoniae directly interferes with HIF-1alpha stabilization in human host cells.. Cell Microbiol.

