# Supplementary figures and images for: CPAF: A Chlamydial Protease in Search of an Authentic Substrate
Source: PLoS Pathog. 2012 Aug 2;8(8):e1002842. doi: 10.1371/journal.ppat.1002842 (PMC3410858; doi:10.1371/journal.ppat.1002842)

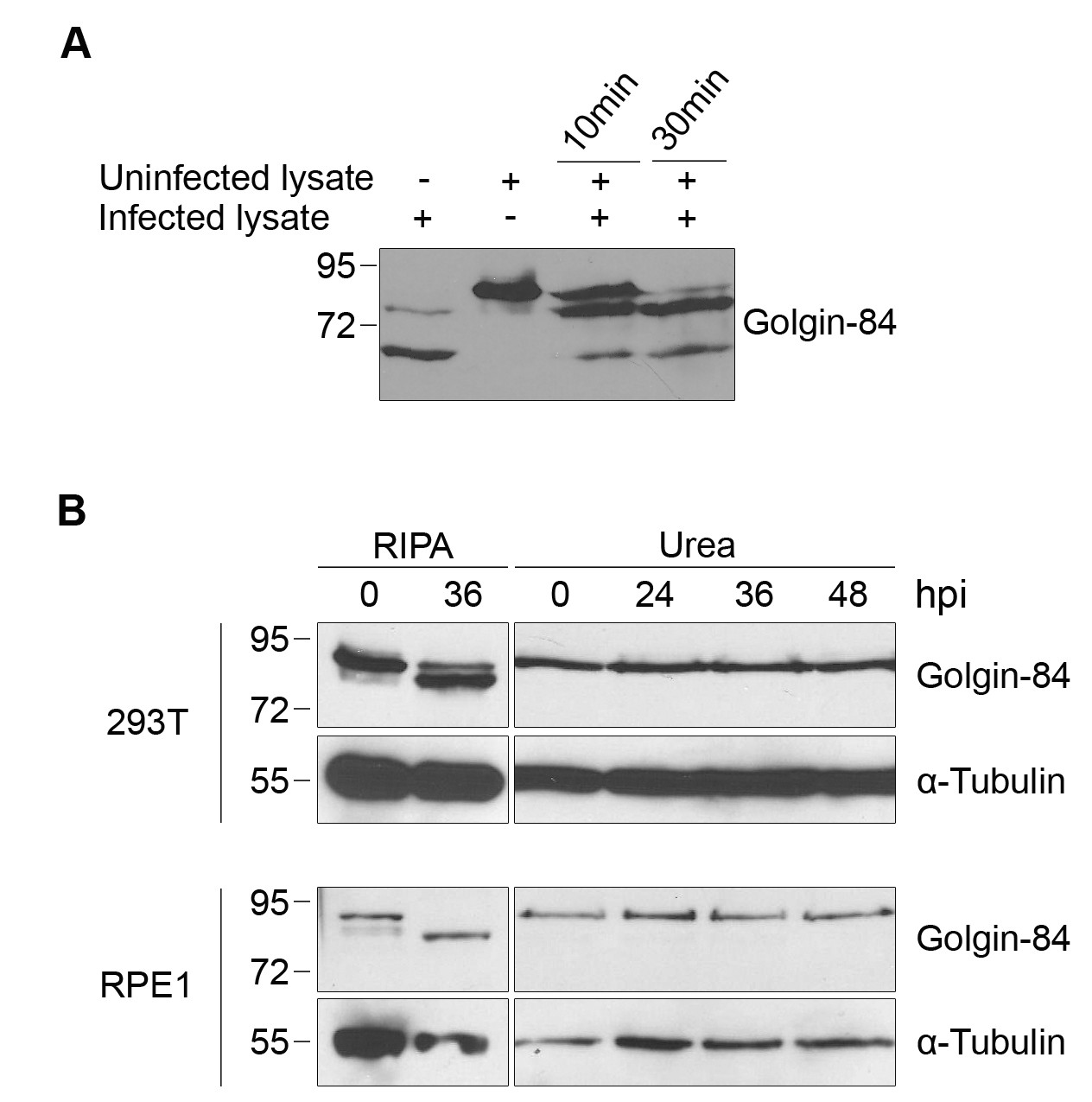

Supplement: Figure S1 — Controls for golgin-84 cleavage experiments in Figure 1 . (A) CPAF is active at 0°C. Cell-free degradation assay in which Chlamydia-infected cell lysate as a source of CPAF was incubated on ice with uninfected cell lysate, as the source of host protein substrates. Reactions were incubated at 0°C for the times indicated and analyzed by immunoblotting with antibodies to golgin-84. (B) Golgin-84 is not cleaved in other Chlamydia-infected cell lines. Lysates of uninfected (0 hpi) or infected HEK 293T (labeled as 293T) and hTERT RPE-1 (labeled as RPE1) cells were prepared in RIPA buffer (left panel) or by direct lysis in 8M urea (right panel) at the indicated times, separated by SDS-PAGE and probed with antibodies to golgin-84 or α-tubulin (loading control). (TIF) [file ppat.1002842.s001.tif]
