# Supplementary material for: CPAF: A Chlamydial Protease in Search of an Authentic Substrate
Source: PLoS Pathog. 2012 Aug 2;8(8):e1002842. doi: 10.1371/journal.ppat.1002842 (PMC3410858; doi:10.1371/journal.ppat.1002842)
Supplement: Table S1 — Antibody information. (DOC) [file ppat.1002842.s002.doc]

**Table S1. Antibody Information**

Western Blot

| **Antibody** | **Species** | **Source** | **Catalog Number** | **Lot Number** | **Transfer Conditions** | **Dilution** | **Incubation Conditions** |
| --- | --- | --- | --- | --- | --- | --- | --- |
| Bim | Rabbit | Sigma | B7929 | 071M1321 | 20% MeOH 80 V 2 hr | 1:1,000 | 1 hr RT |
| Puma | Rabbit | Sigma | P4618 | 099K1254 | 20% MeOH 80 V 2 hr | 1:1,000 | 1 hr RT |
| Keratin-8 | Mouse | Sigma | C5301 | 028K4762 | 20% MeOH  100V 1hr | 1:1,000 | 1hr RT |
| Keratin-18 | Mouse | Sigma | C1399 | 080M4787 | 20% MeOH  15V 1hr (Semi-Dry) | 1:1,000 | 1hr RT |
| α-Tubulin | Mouse | Sigma | T5168 | 078K4781 | 20% MeOH  100V 1hr | 1:10,000 | 1hr RT |
| Vimentin | Mouse | Sigma | V5255 | 099K4753 | 20% MeOH  100V 1hr | 1:1,000 | 1hr RT |
| Cyclin-B1 | Rabbit | Santa Cruz | Sc-752 | L1409 | 20% MeOH  100V 1hr | 1:1,000 | 1hr RT |
| Nectin-1 | Mouse | Santa Cruz | Sc-21722 | H2708 | 20% MeOH  100V 1hr | 1:1,000 | 1hr RT |
| P65 | Mouse | Santa Cruz | Sc-8008 |  | 20% MeOH  100V 1hr | 1:1,000 | 1hr RT |
| Bik | Rabbit | Cell Signaling | 4592S | 3 | 20% MeOH 80 V 2 hr | 1:1,000 | O/N 4°C |
| Erk 1/2 | Mouse | Cell Signaling | 9107 | 4 | 20% MeOH  100V 1hr | 1:15,000 | 1hr RT |
| Caspase-3 | Mouse | BD Biosciences | 610322 | 51345 | 20% MeOH  100V 1hr | 1:1,000 | 1hr RT |
| RFX5 | Rabbit | Rockland Immuno-chemicals | 200-401-194 | 14562 | 20% MeOH  100V 1hr | 1:1,000 | 1hr RT |
| CPAFc | Mouse | Gift from Dr. Guangming Zhong |  |  | 20% MeOH  100V 1hr | 1:100 | 1hr RT |
| Golgin-84 (head) | Sheep | Gift from Dr. Martin Lowe |  |  | 20% MeOH  100V 1hr | 1:1,000 | 1hr RT |
| MOMP (VD4 epitope) | Mouse | Gift from Dr. Ellena Peterson |  |  | 20% MeOH  100V 1hr | 1:1,000 | 1hr RT |

Immunofluorescence

| **Antibody** | **Species** | **Source** | **Catalog Number** | **Lot Number** | **Fixation** | **Concentration** | **Incubation Conditions** |
| --- | --- | --- | --- | --- | --- | --- | --- |
| Vimentin | Rabbit | Cell Signaling | 5741P | 1 | 4% Formaldehyde | 1:100 | O/N 4°C |
| Mannosidase II | Rabbit | Gift from Dr. Kelley Moremen |  |  | 4% Formaldehyde | 1:1,000 | 1hr RT |
| MOMP (VD4 epitope) | Mouse | Gift from Dr. Ellena Peterson |  |  | 4% Formaldehyde | 1:1,000 | 1hr RT |
